# RELATIONSHIPS BETWEEN INFORMAL CAREGIVERS’ MARITAL SATISFACTION, BURDEN, AND CAREGIVING STRESS

**DOI:** 10.1093/geroni/igz038.1058

**Published:** 2019-11-08

**Authors:** Hannah Ottmar, Amy Houston, Elisabeth Harfmann, Amy Olzmann, Glenna Brewster

**Affiliations:** 1 Xavier University, Cincinnati, Ohio, United States; 2 Milwaukee VAMC, Milwaukee, Wisconsin, United States; 3 Baltimore VAMC, Baltimore, Maryland, United States; 4 Emory University Nell Hodgson Woodruff School of Nursing, Atlanta, Georgia, United States

## Abstract

Spouses often serve as the primary caregiver of individuals with dementia (Wright, 1991). As caregiving can be a stressful experience, marriage strains may occur. Researchers have found that caregiver spouses reporting low marital cohesion and satisfaction endorsed significant symptoms of depression (Rankin et al., 2001). Lower levels of marital intimacy have been found to be associated with higher levels of depression and strain among caregivers (Morris et al., 1998). The goal of the current study was to better understand the relationships between caregiver (N = 158) marital satisfaction, caregiving stress, and subjective and objective burden. Results indicated that marital satisfaction was a significant negative predictor of subjective burden as measured by the Zarit Burden Interview (F(1,134) = 93.51, p < .001, R2 = .411), subjective burden as measured by the Revised Memory and Behavior Problems Checklist (RMBPC), Reaction Scale (F(1, 146) = 25.65, p < .001, R2 = .149), and role captivity (F(1, 139), P < .001, R2 = .380). Marital satisfaction was a significant positive predictor of caregiver competence (F(1,140) = 32.45, p < .001, R2 = .188). It was also found that marital satisfaction was not a significant predictor of objective burden as measured by the RMBPC Frequency Scale (F(1, 146), p = .065, R2 = .023). The findings have implications for future interventions in that improving marital satisfaction of spouse caregivers may reduce subjective burden, decrease feeling trapped within the caregiving role, and increase caregivers’ sense of competence within the caregiving role.

